# Comparison of survival, function and complication between intercalary frozen autograft versus massive allograft reconstruction after malignant bone tumors resection

**DOI:** 10.1186/s10195-024-00807-w

**Published:** 2024-11-24

**Authors:** Zhuoyu Li, Haoyu Guo, Zhiping Deng, Yongkun Yang, Qing Zhang, Weifeng Liu

**Affiliations:** 1 Department of Orthopaedic Oncology Surgery, Beijing Jishuitan Hospital, Capital Medical University, Beijing, 100035 China; 2 National Center for Orthopedics, Beijing, 100035 China; 3 Beijing Research Institute of Traumatology and Orthopaedics, Beijing, 100035 China

**Keywords:** Intercalary biological reconstruction, Allograft, Frozen autograft, Graft survival, Complications

## Abstract

**Purpose:**

This study aims to compare the clinical outcomes of intercalary frozen autograft and allograft reconstruction for primary malignant bone tumors.

**Methods:**

A retrospective study was conducted on 144 patients who underwent intercalary biological reconstruction for primary malignant bone tumors at a single institution between January 2012 and July 2023. Seventy-two patients underwent intercalary liquid nitrogen-frozen autograft reconstruction, and 72 patients underwent intercalary allograft reconstruction in this study. A modified International Society of Limb Salvage classification system was used to evaluate the complications.

**Results:**

The mean follow-up time was 60.2 ± 32.1 (range, 12–149) months. The mean union time was 9.6 months in the frozen autograft group and 15.9 months in the allograft group (*p* < 0.001). The 5-year overall survivorship was 86.8% in the frozen autograft group and 73.2% in the allograft group (*p* = 0.017). The average MSTS-93 score was comparable between the two groups (89.7% by autograft versus 87.6% by allograft, *p* > 0.05). Of the patients, 48.6% (70/144) had at least one complication. The most common complications were bone nonunion (20.8%, 30/144), followed by structural failure (17.4%, 25/144), tumor progression (10.4%, 15/144), infection (10.4%, 15/144), and soft tissue failures (5.6%, 8/144). Higher rates of bone nonunion (type 4B; *p* = 0.002) and structural failure (type 3B; *p* = 0.004) were obtained in the allograft group than in the frozen autograft group.

**Conclusions:**

The intercalary frozen autografts had shorter union time and lower complication rates than allograft reconstruction. Therefore, we recommend that frozen autograft reconstruction be considered when the tumor bone has not suffered severe osteolytic injury or pathological fracture.

*Level of evidence*: level III, case–control study.

## Introduction

With the development of chemotherapy and limb reconstruction techniques, the majority of patients with bone tumors underwent limb-sparing surgery [1, 2]. However, intercalary reconstruction of bone defects after tumor resection remains challenging. As the periarticular muscle attachment points and ligaments are preserved, patients may obtain better proprioception and joint function [3]. Intercalary reconstruction techniques have been widely reported, including intercalary endoprosthesis, allograft, autograft, vascularized fibula, allograft prosthetic composites, bone transport techniques, and membrane induction techniques [4–8]. Mechanical reconstruction is the most widely used technique for bone defect reconstruction worldwide and provides immediate stability and the fastest return to weight bearing; however, it also has a high incidence of prosthetic loosening and long-term failure [4].

Biological reconstruction has the advantage of preserving bone volume and long-term reconstructive effectiveness [9]. Allografts are the most commonly used biologic reconstruction technique and can provide satisfactory long-term functional results, but the incidence rates of infection, fracture, and nonunion are quite high [10]. Inactivated autografts are commonly used for reconstruction in East and Southeast Asian countries and have the advantages of good structural match, low cost, no need for bone banking, and no transmission of disease [5]. There is no consensus on the best technique to inactivate autografts, and Takeuchi et al. recently reported similar autograft survival and complication rates with freezing, radiation, and pasteurization [11]. Previous small case series studies have compared differences in outcomes between allograft and autograft reconstruction after extensive tumor resection [12]. However, studies directly comparing intercalary allografts with inactivated autografts are scarce.

The purpose of this study was to compare the differences in clinical outcomes between frozen autograft and allograft reconstruction and to answer the following questions: (1) What is the difference in graft survival between patients who accept frozen autograft and allograft reconstruction? (2) What is the difference in the incidence of transplant-related complications between patients who accept frozen autograft and allograft reconstruction? (3) Is there a difference in function in patients retaining frozen autograft and allograft reconstruction at last follow-up?

## Patients and methods

We retrospectively reviewed the clinical data of 163 patients who underwent intercalary frozen autograft or massive allograft reconstruction, which was prospectively collected at our institution between January 2012 and July 2023 (Table 1). This study was approved by the Ethics Committee of Beijing Jishuitan Hospital. Intercalary reconstruction was defined as a segmental tumor resection that did not involve the joint. Cases reconstructed by allograft or autograft combined with vascularized fibular flaps or any hybrid reconstruction methods in initial procedures were not included in this study. Patients who underwent intercalary frozen autograft or allograft reconstruction were followed up for at least 1 year. Patient demographics, radiological outcomes, graft survival, and graft-related complications were recorded. Nineteen patients (11.6%) were excluded owing to loss of follow-up or incomplete datasets.

**Table 1:** Patient demographics of all patients

Variable	Total (*n*, range, %)	Autograft (*n*, range, %)	Allograft (*n*, range, %)	*P* value
n	144	72	72	
Length (mean, cm)	16.9 (8–34)	17.9 (9–34)	15.8 (8–31)	0.682
Gender				0.313
Male	112 (7–73)	44 (61%)	38 (53%)	
Female	96 (12–74)	28 (39%)	34 (47%)	
Age (mean, years)	23.8 (6–64)	22.0 (6–61)	25.6 (7–64)	0.437
Tumor types				0.759
Osteosarcoma	91 (63%)	50 (70%)	44 (57%)	
Ewing sarcoma	13 (9%)	9 (13%)	7 (7%)	
Adamantinoma	12 (8%)	7 (10%)	5 (7%)	
Chondrosarcoma	10 (7%)	7 (10%)	7 (10%)	
Others	18 (12%)	5 (7%)	9 (13%)	
Grade				0.843
Low	31 (23%)	16 (22%)	15 (21%)	
High	111 (58%)	56 (78%)	57 (79%)	
Resected area				0.240
Femur	72 (50%)	39 (54%)	33 (46%)	
Tibia	51 (35%)	26 (36%)	25 (35%)	
Humerus	21 (15%)	7 (10%)	14 (19%)	
Reconstruction				0.104
Plate	101 (70.1%)	57 (79.2%)	44 (61.1%)	
Plate + IN	15 (10.4%)	6 (8.3%)	9 (6.3%)	
IN	28 (19.4%)	10 (13.9%)	18 (12.5%)	
Chemotherapy				0.189
Yes	105 (73%)	56 (78%)	49 (60%)	
No	39 (27%)	16 (22%)	23 (40%)	
Radiotherapy				0.698
Yes	7 (5%)	4 (6%)	3 (4%)	
No	137 (95%)	68 (94%)	69 (96%)	
Follow-up (mean, months)	60 (12–149)	56 (12–100)	64 (12–149)	0.536

*IN* intramedullary nailing

A total of 144 patients were included in the study, of whom 72 underwent intercalary allograft reconstruction and 72 underwent intercalary frozen autograft reconstruction.

The mean age of the patients was 23.8 ± 14.3 (6–64) years (22.0 years in autograft group versus 25.6 years in allograft group, *p* = 0.437). There were 82 male (57%) and 62 female (43%) patients. There were 44 male and 28 female patients in the autograft group, whereas there were 38 male and 34 female patients in the allogeneic group (*p* = 0.313). The most common tumor type was osteosarcoma (91 cases), followed by Ewing sarcoma (13 cases), adamantinoma (12 cases), chondrosarcoma (10 cases), undifferentiated pleomorphic sarcoma (UPS) of the bone (8 cases), hemangiosarcoma of the bone (5 cases), spindle cell sarcoma of the bone (3 cases), malignant giant cell tumor of the bone (1 case), and malignant giant cell tumor of the tendon sheath (1 case). The results showed 78% of all cases were (56/72) high-grade sarcomas in the autograft group and 79% (55/72) were high-grade sarcomas in the allograft group. Of the patients, 50% (72/144) underwent femoral reconstruction, 35% (51/144) underwent tibial reconstruction, and 15% (21/144) underwent humeral reconstruction. No difference was found between the two groups in terms of reconstruction site (*p* = 0.240). Of the patients, 69% (99/144) received chemotherapy and 7 (5%) received radiotherapy. The mean reconstructed length was 16.9 ± 6.3 cm (17.9 cm in autograft group versus 15.8 years in allograft group, *p* = 0.682). Of the patients, 75% (107/144) underwent plate fixation, 15% (22/144) underwent intramedullary nailing combined with plate fixation, and 10% (15/144) underwent intramedullary nailing fixation only. Of the patients, 71% (102/144) underwent metaphyseal reconstruction and 29% (42/144) underwent diaphysis reconstruction (Table 1).

The surgical technique has been described in previous studies [13, 14]. All patients underwent en bloc resection of the bone tumor. For frozen autograft reconstructions, the attached soft tissue was excised and the intramedullary contents was removed using a reamer or curette. We use a 4-mm-diameter Stern’s needle to perforate the cortex along the long axis of the bone at intervals of 2 cm to prevent fracture during liquid-nitrogen freezing. The bone was immersed in liquid nitrogen for 30 min, thawed at room temperature (25 ℃) for 15–20 min, and rewarmed in saline for 15 min. All allografts were harvested and packaged under sterile conditions and stored frozen at −80 °C in the bone bank that is established at our institution according to a technique that has been previously described [13]. Several reconstruction techniques were used to fix the grafts (Table 1). In most cases, we used intramedullary bone cement combined with bilateral bridging plates to fix the grafts, while some patients accepted intramedullary nailing fixation only or intramedullary nailing combined with plate fixation.

The primary study endpoint in this study was graft survival, and graft failure was defined as graft removal for any reason. The secondary study endpoint was the complication rate, and complications were recorded according to the modified Henderson classification proposed by the International Society for Limb Salvage [15]. Bone union was confirmed when more than 75% of the cortex at the junction bridged to the frozen graft at the host bone diaphysis in both X-ray views (anteroposterior (AP) or lateral view) or any computed tomography (CT) plane. Union time of metaphyseal and diaphyseal osteotomy sites was recorded. Delayed union was defined as union 2 years after surgery. Nonunion was defined as no partial bony junction between host bone and grafts 2 years postoperatively or additional surgery for promoting union at the graft–host junction. The graft fracture was defined as a fracture away from the graft–host bone junction. We used the Musculoskeletal Tumor Society score (MSTS-93) to evaluate function, which was assessed only in patients who retained their frozen autografts or allografts at last follow-up [16]. A total of 112 patients were available for functional evaluation at last follow-up.

### Statistical analysis

Bivariate analysis was done with the Student *t*-test or chi-square test. The Kaplan–Meier method and log-rank test were used to analyze the difference in union time and graft survival, including revision for any reason and removal of graft as endpoints indicating failure. All tests were two-sided, and *p*-value < 0.05 was considered significant. Bonferroni correction for multiple comparisons was applied by dividing the significance level by the number of tests performed. Statistical analysis was performed using Statistical Package for the Social Sciences (SPSS) software version 26.0 (IBM, USA) and GraphPad Prism software version 10 (USA).

## Results

The demographic data and surgical details of all patients are presented in Table 1. The mean follow-up time was 60.2 ± 32.1 (range, 12–149) months. Of the patients, 59% (85/144) were followed up for more than 5 years. At last follow-up, 24 patients had died of the disease, 16 patients were alive with evidence of disease, and 104 patients were alive with no evidence of disease. Fifteen patients had local recurrence, and 35 patients had distant metastases (21 lung metastases, 8 lung and bone metastases, and 6 bone metastases).

Of all patients included in this study, host–graft bone united primarily in 112 cases (65 cases in the frozen autograft group and 47 cases in the allograft group). The mean host–graft bone union time was 9.6 ± 4.8 (6–27) months in the frozen autograft group and 15.9 ± 6.7 (8–37) months in the allograft group (*p* < 0.001).

**Fig. 1:**
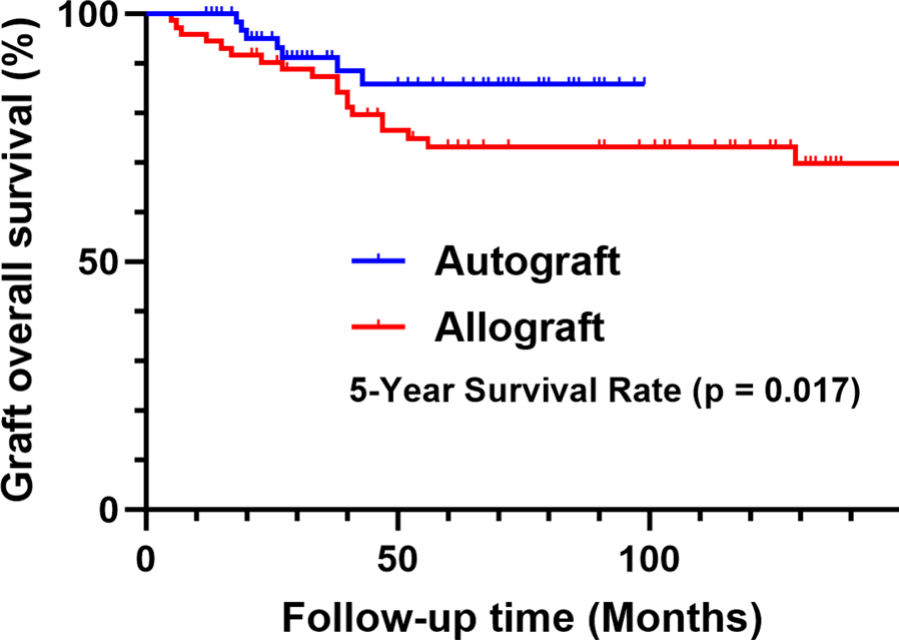
Kaplan–Meier survival curve showing the overall survival of the intercalary allograft and autograft reconstruction (log-rank test)

The 5-year overall survival rate for frozen autografts was 86.8% and for allografts was 73.2% (*p* = 0.017, Fig. 1). Of the seven patients who had complications leading to graft removal in the frozen autograft group, six cases were due to local recurrence and one was due to deep infection. However, of the 19 patients who underwent graft removal in the allograft group, 7 cases were due to infection, 5 to local recurrence, 4 to bone nonunion, and 3 to structural failure (Table 2).

**Table 2:** All causes for frozen autograft and allograft removal

Cause of graft removal	Frozen autograft (*n* = 72)	Allograft failure (*n* = 72)	*p*
Soft tissue failure	0 (0.0%)	0 (0.0%)	–
Nonunion	0 (0.0%)	4 (5.6%)	0.043^✱^
Structural failure	0 (0.0%)	3 (4.2%)	0.080
Infection	1 (1.4%)	7 (9.7%)	0.029^✱^
Tumor progression	6 (8.3%)	5 (6.9%)	0.754

^✱^*p* < 0.05, indicating a significant difference between the two groups

According to the modified Henderson classification, 48.6% (70/144) of patients had at least one complication. The most common complications were bone nonunion (20.8%, 30/144), followed by structural failure (17.4%, 25/144), tumor progression (10.4%, 15/144), infection (10.4%, 15/144), and soft tissue failures (5.6%, 8/144). Higher rates of bone nonunion (type 4B; *p* = 0.003) and structural failure (type 3B; *p* = 0.012) were obtained in the allograft group than in the frozen autograft group (Table 3).

**Table 3:** Graft failure modes according to the modified International Society of Limb Salvage (ISOLS) classification system

Complications	Subcategory	Frozen autograft (*n* = 72)		Allograft (*n* = 72)		P
		*n*	%	*n*	%	
Soft tissue failure	1A functional	0	0.0	0	0.0	–
	1B coverage	3	4.2	5	6.9	0.735
Nonunion	2A hypertrophic	0	0.0	2	2.8	0.336
	2B atrophic	7	9.7	21	29.2	0.002^✱^
Structural failure	3A fixation	6	8.3	8	11.1	0.621
	3B graft	1	1.4	10	13.9	0.004^✱^
Infection	4A early	1	1.4	3	4.2	0.183
	4B late	3	4.2	8	11.1	0.076
Tumor progression	5A soft tissue	6	8.3	6	8.3	0.823
	5B host bone	1	1.4	2	2.8	0.508
	5C graft	0	0.0	0	0.0	–

^✱^*p* < 0.05, indicating a significant difference between the two groups

**Fig. 2:**
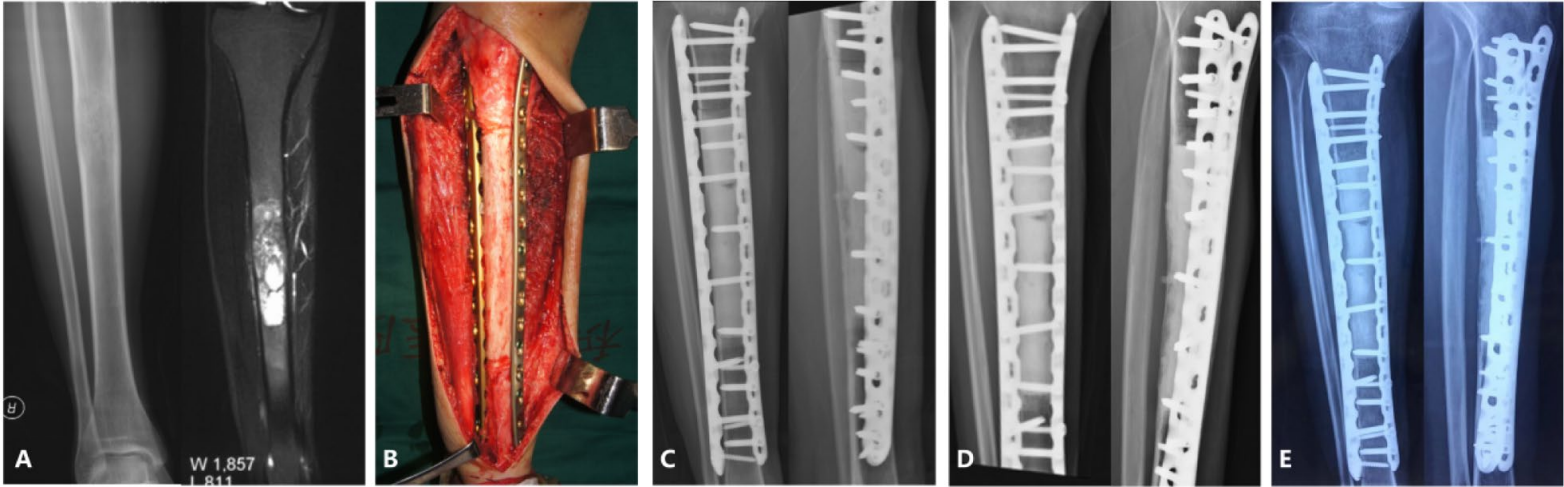
A 30-year-old male with Ewing sarcoma of the right tibia. **A** X-ray and T2-weighted MRI showed tumor was located in the right tibial diaphysis. **B** The autogenous bone was inactivated in liquid nitrogen and reconstructed with bilateral bridging plates. **C** Immediate postoperative image shows bridging plate fixation combined with intramedullary cement. **D** At 10 months postoperatively, the proximal and distal osteotomy sites had excellent consolidation. **E** Five years after surgery, the autograft is still in good condition

**Fig. 3:**
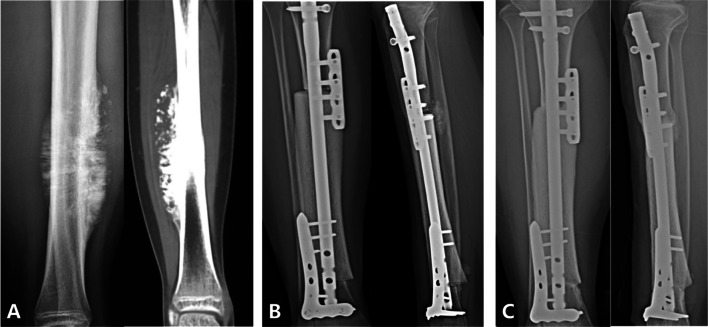
A 14-year-old female with osteosarcoma of the right tibia. **A** X-ray and CT scan showed tumor was located in the right tibial diaphysis. **B** The allograft bone was implanted and reconstructed with intramedullary nailing and two short plates. **C** At 25 months postoperatively, the proximal and distal osteotomy sites had excellent consolidation

Functional outcomes were obtained in 51 patients who underwent allograft reconstruction and 61 patients who underwent autograft reconstruction. The mean MSTS-93 score was 89.7% for autograft reconstruction and 87.6% for allograft reconstruction (*p* > 0.05). Patients reconstructed with plates or intramedullary nail fixation combined with plates had a mean MSTS-93 score of 89.3%, while the MSTS-93 score was 83.0% in patients reconstructed with intramedullary nail fixation only (*p* = 0.02). The patients with tibial tumor resection and reconstruction had a trend of higher MSTS-93 score (88.1%) than those in the femur (84.5%), while there was no statistical difference (*p* = 0.212, Fig. 2 and Fig. 3).

## Discussion

Allografts and inactivated autografts are widely used biological reconstruction techniques [2, 3, 5, 9–11]. The most common complications of both reconstruction techniques are bone nonunion, fracture, and infection [9]. Previous studies directly comparing the clinical outcomes of allograft and autograft reconstruction are scarce [12, 17]. Chen et al. reported the clinical outcomes of allograft and autograft using multiple reconstruction techniques, showing comparable union time and clinical outcomes [17]. However, that study included multiple reconstruction modalities such as allograft-prosthesis composites, intercalary reconstruction, arthrodesis, osteoarticular, and hemicortical reconstruction. Different reconstruction modalities may have different complications, and graft survival may vary between reconstruction techniques, therefore we compare the clinical outcome differences between the two intercalary biologic reconstructions applied in our institution.

We found that the 5-year survival rate of frozen autografts was higher than that of allografts, which may be because allografts have a higher rate of graft failure, nonunion, and infection. We hypothesize that these complications are due to graft integration and simultaneous soft tissue and vascular growth in the first few years after reconstruction. According to the modified ISOLS classification, our study showed a higher complication rate for allograft (61.1%) compared with frozen autograft reconstruction (33.3%). Chen et al. also reported a higher complication rate for allografts, but their study had a lower overall complication rate in both autograft (14.6%, 24/164) and allograft reconstruction (30.8%, 28/91) [17]. The use of fresh allografts was the possible reason for the lower complication rate. They used fresh-frozen allografts for reconstruction, compared with our bone bank’s deep cryopreservation conditions, and previous studies have shown that long-term deep cryopreservation (−80 °C) of allografts may increase the probability of contamination, and the biological activity of bone morphogenetic proteins (BMPs) decreases over time. Frozen autografts underwent immediate intraoperative tumor inactivation and reconstruction, which may better preserve the osteoinductive and osteoconductive capacity of the graft bone.

In our series, the common complications were similar in both groups, including bone nonunion, structural failure, infection, and tumor progression, which is similar to former studies [2, 3, 5, 9–11, 18, 19]. The rate of local tumor recurrence in host bone and soft tissue was similar for both grafts (11.1% for allografts and 9.7% for autografts). Although recurrence was one of the main factors for graft failure in both groups, all recurrences were due to positive margins in soft tissue or host bone, and no graft-related local recurrences were found. We therefore believe that local recurrence was not a key factor in the comparison between the two groups, as it was related to resection rather than reconstruction.

Our results show that allografts have a longer union time and a higher nonhealing rate than frozen autografts. A meta-analysis showed that irradiated and frozen autograft bone had a lower nonunion rate than allograft-inactivated bone [20]. However, Chen et al. reported similar healing times and nonunion rates for allografts (8.4 months, 13.3%) and autografts (9.5 months, 11.6%) [17]. Our study showed similar healing times for autologous recycled grafts, but longer healing times for allografts. The reason for the shorter healing time of allograft bone in their study may be that all fresh allograft bone was used, whereas deep cryopreserved allografts were used in our center. The bioactivity of bone morphogenetic proteins (BMPs) in allografts decreases with longer storage time, which may affect the healing between the host bone and the graft.

Our study shows that frozen autografts have a lower rate of graft fracture, a finding supported by several case series and systematic reviews. We suggest that the process of vascularization of allografts relies on the colonization of the host’s living cells on the surface of the allograft bone to produce osteoclasts for bone resorption, which reduces the mechanical strength of the allograft and thus increases the fracture rate. In addition to this, bone morphogenetic protein (BMP) bioactivity was higher in frozen autografts than in allografts, which further explains the slowness of the bone remodeling process in allografts. Several techniques have been reported to enhance the structural strength of recycled autografts or allografts. Gupta et al. reported a low fracture rate (4.3%) for intercalary allograft reconstruction augmented by intramedullary cement combined with plate fixation [21]. A meta-analysis by Huang et al. also supported these findings [20]. We believe that bridging plate fixation may provide stronger fixation before healing of the host bone and graft, which reduces fractures due to nonunion to some extent, while intramedullary cement may enhance the mechanical strength of the graft, thus further reducing the fracture rate.

However, our results showed differences in the causes of graft removal between the two groups. The most common cause of graft removal in allografts was deep infection, whereas the most common cause of removal in autografts was local progression. Previous studies have shown a 0–18% incidence of infection in allografts, which is comparable to our results [2, 3, 9, 10, 17]. The incidence of late infection was lower for autografts than for allografts owing to the higher risk of contamination in the handling and long-term storage of allografts. In addition to this, we found similar rates of local recurrence and graft removal due to local progression between the two groups. We did not observe any cases of local recurrence originating from regenerative autografts in patients who received frozen autografts. Therefore, we consider the similar local recurrence rates in both groups to be reasonable.

We found that patients who underwent intercalary frozen autografts (89%) had similar functional scores to those who underwent intercalary allograft reconstruction (87%), which is similar to previous reports in the literature [2, 3, 5, 9–11]. These satisfactory functional outcomes may be because the intercalary reconstruction preserved the joint ligaments and muscle attachment points. We excluded patients with graft removal or amputation when evaluating functional outcomes, so there may be an overestimation of functional scores. However, we believe that this comparison is still meaningful considering that we adopted the same comparison strategy between the two groups.

This study also has some limitations. First, we did not compare the difference in outcomes between the two modalities using osteoarticular reconstruction. In our experience, intercalary reconstruction that preserves the native joint and muscle stops has an advantage. Second, allografts and autografts have a high complication rate in osteoarticular reconstruction. In addition, we only compared the functional outcomes of patients with preserved allografts or autografts at the last follow-up and did not include patients with amputations and arthroplasties, which may lead to an overestimation of patients’ functional outcomes. Our aim was to assess functional outcomes in patients with preserved biologic reconstruction, which did not apply to these patients. Finally, our use of only single-center data may limit the representativeness of the results. Future studies should be conducted prospectively to eliminate the effect of patient selection bias on outcomes.

In conclusion, our large retrospective study compares the reconstructive results of intercalary allografts with frozen autografts after resection of primary malignant bone tumors in long bones. Frozen autografts had shorter healing times and lower complication rates compared with allografts. Therefore, we suggest that frozen autografts should be considered for extremity reconstruction when the tumor bone has not suffered severe osteolytic injury or pathological fracture.

## Data Availability

All data used in this study are not publicly available because of patient confidentiality but are available from the corresponding author on reasonable request.
